# Medical students’ perception of assessment and its effects on their learning in Dubai: a convergent mixed methods study

**DOI:** 10.3389/fmed.2025.1620437

**Published:** 2025-08-04

**Authors:** Eman Khalaf, Farah Ennab, Farah Otaki, Shaista Salman Guraya, Fatemeh Amir-Rad, Erum Khan, Ritu Lakhtakia

**Affiliations:** ^1^Al Amal Psychiatric Hospital, Emirates Health Services, Dubai, United Arab Emirates; ^2^Institute of Learning (IoL), Mohammed Bin Rashid University of Medicine and Health Sciences (MBRU), Dubai Health, Dubai, United Arab Emirates; ^3^Strategy and Institutional Excellence (SIE), Mohammed Bin Rashid University of Medicine and Health Sciences (MBRU), Dubai Health, Dubai, United Arab Emirates; ^4^Department of Health Services Research, Faculty of Health, Medicine, and Life Sciences (FHML), Care and Public Health Research Institute (CAPHRI), Maastricht University, Maastricht, Netherlands; ^5^Hamdan Bin Mohammed College of Dental Medicine (HBMCDM), Mohammed Bin Rashid University of Medicine and Health Sciences (MBRU), Dubai Health, Dubai, United Arab Emirates; ^6^School of Medicine, Dentistry and Biomedical Sciences, Queen’s University Belfast, Belfast, United Kingdom; ^7^College of Medicine, Ajman University, Ajman, United Arab Emirates; ^8^College of Medicine (CoM), Mohammed Bin Rashid University of Medicine and Health Sciences (MBRU), Dubai Health, Dubai, United Arab Emirates

**Keywords:** assessment, Self-regulated learning, mixed methods, health professions’ education, Dubai, United Arab Emirates Sustainable Development Goals, SDG 3, SDG 4

## Abstract

**Background:**

Enhanced learning is achieved when assessments are effectively designed in alignment with the learning objectives and supported by ongoing research in the field. Although it is universally acknowledged that assessments are essential in medical education, little is known about assessment policy and characteristics, and its influence on learning and teaching in medical schools in the Middle East and North Africa region (MENA). The purpose of this study is to investigate the perception of medical students of the assessment method implemented in a medical school in Dubai, United Arab Emirates.

**Methods:**

A convergent mixed methods study design was employed. Quantitative and qualitative data were independently collected and analyzed. The quantitative component comprised a cross-sectional observational survey design where a tailormade survey with a five-point Likert-type scale was administered to 87 undergraduate medical students. The corresponding quantitative data was analyzed using the Statistical Package for Social Sciences (SPSS, version 27.0). As for the qualitative data, it was collected through a series of focus group sessions aimed at exploring students’ perception of assessment and its impact on their learning. The corresponding analysis was inductive, following the six-step approach introduced by Braun and Clarke. Following that, merging of the information brought about from the two sources generated meta-inferences, which raised the validity of the study’s findings.

**Results:**

The percentage of the total average of agreement of the current assessment method efficacy, according to a tailormade survey protocol of 28 components, measured using a five-point Likert-type scale, was 62.24%. This percentage was calculated by dividing the overall mean (i.e., 87.13) by 140 since it is the maximum possible value (i.e., five of the Likert-type scale multiplied by 28 components) and multiplying it by 100. The inductive thematic analysis of the data, collected via focus group sessions, yielded a novel conceptual framework: “Medical Students” Take on Assessment Method’, with two overarching themes: Development Process and Consequences. Within the Development Process theme, two categories emerged: Assessment plan and Student support. As for the Consequences theme, it included three other categories: Output, Outcomes, and Impact. Lastly, the following four meta-inferences emerged from integrating the quantitative with the qualitative analysis findings: Processual perspective, Learners’ reaction, Inclusiveness, and Ripple effects.

**Conclusion:**

This study reinforced the importance of effectively assessing medical students’ competences while maximizing the learning value of the encapsulating assessment method. It showed that it is not about making choices around discrete aspects of assessment, but rather to consider an assessment method holistically as dynamic processes with several moving parts. Ideally, assessment methods should be designed, implemented, and maintained in ways that would maximize their learning value, taking into account the corresponding context and learners’ perception.

## 1 Introduction

Assessments constitute the means by which competences are measured, determining whether, or not, learning outcomes have been achieved. They form an integral part of learning and teaching in general, and medical education more specifically (1, 2). While assessments can bring the learners a sense of achievement and confidence, they can also induce stress and anxiety, and even sometimes loss of faith in one’s capabilities (3, 4). Assessments have always been an important part of the education process and students’ progression worldwide. This is particularly relevant in medical schools where the assessment of knowledge, skills, and attitudes plays a crucial role in graduating safe and competent, and socially responsible doctors (5). Research is increasingly emphasizing the role of assessments in measuring and also improving students’ academic performance (6–13). Assessment also acts as a tool for assessors to obtain insight into the delivered content and in turn developed competencies. Educators can leverage assessment as a quality tool to confirm that what they had intended to deliver, through their teachings, actually went across and in turn got integrated (as competencies) by the learners (14–16). Assessors often utilize assessment as a reflection tool to trace trends in students’ performance and in turn identify opportunities for continuous improvement of learning and teaching (altogether, including but not limited to the assessments themselves) (17). The importance of assessments in medical schools has been even more pronounced in recent years (18). A lot of advancements have been made to make the measurement of practical competencies on a par with that of the theoretical ones (19). The entailed developments are strengthening the assessment of both basic and clinical medical sciences competencies, along with the necessary integration across them. Moreover, it is established that the choice of assessment formats affects the learning strategies that students deploy and in turn their capacity to maximize their experiences as part of medical programs (20, 21).

Systematically capturing and analysing students’ perceptions of the assessment method enables medical educators to continuously adapt and improve their approaches (22). Enhanced learning is achieved when assessments are carefully designed in alignment with the learning objectives and supported by ongoing research in the field (23). The learning experience is shaped by a sophisticated interplay of many variables, including the students’ academic performance and their individual knowledge processing capacity; hence, it crucial to be systemic in the means by which the students’ perception is captured and interpreted (24). Although it is universally acknowledged that assessments are essential in medical education (25–27), little is known about students’ perception of assessment policy and characteristics (28–30), and its influence on learning and teaching in medical schools in Middle East and North Africa region (MENA). Therefore, the overall purpose of this study is to investigate the perception of medical students of the assessment method implemented in a medical school in Dubai, United Arab Emirates (UAE). The mixed methods research design adapted for the current study is meant to answer the following three questions, corresponding as per established mixed methods research reporting standards (31) to the quantitative, qualitative, and integration components, respectively:

1. To what extent do the students agree about the appropriateness of specific assessment method attributes, and to what extent did the students’ demographic characteristics affect their perception of the assessment method in place?
2. What do the students think of the purpose, extent of accommodation, relevance, and structure of the assessment method in place?
3. What aspects of the students’ perception need to be taken into account to maximize the learning gained through assessment processes?

## 2 Material and methods

### 2.1 Context of the study

**FIGURE 1:**
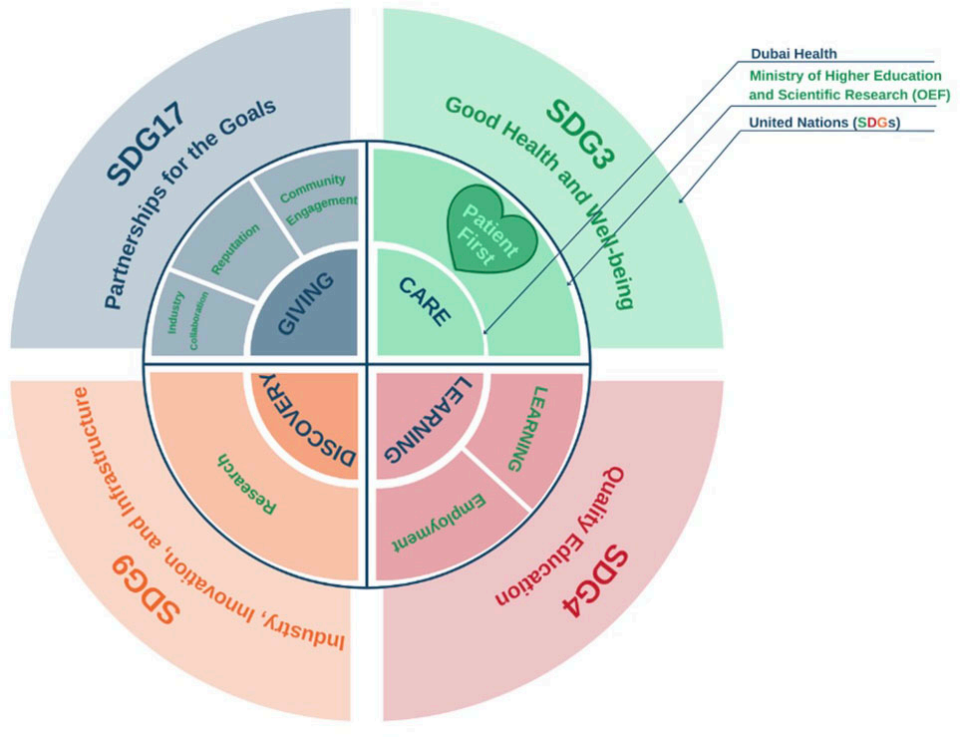
Alignment of goals (micro-, meso-, and macro-levels), as illustrated in another mixed methods investigation that took place in the same context of the current study (37). This figure shows how the pillars of Dubai Health (Care, Learning, and Discovery, and Giving) are feeding into the pillars of the ministerial Outcome-based Evaluation Framework (OEF) which in tun contribute to select Sustainable Development Goals (SDGs). Patient first, as the core value of Dubai Health [purposely located in the heart of the illustration, slightly toward the left (in Green, which is considered a balanced anchor for the other colors of the visible spectrum)]. This patient centricity is among the key differentiators of MBRU from the rest of the higher education institutions governed by the OEF of the UAE Ministry of Higher Education and Scientific Research.

This study was conducted at Mohammed Bin Rashid University of Medicine and Health Sciences (MBRU) in Dubai Health in Dubai, UAE (32). MBRU is the implementation vehicle of two pillars (i.e., “learning” and “discovery”) of the first integrated academic health systems in Dubai, namely: Dubai Health (33). Other than its “learning” and “discovery” pillars, Dubai Health oversees the operations of around 40%–60% of the Dubai health sector through its clinical enterprise (i.e., “care” pillar). Besides, the “care,” “learning,” and “discovery” pillars, that can be mapped onto the traditional tripartite mission of typical academic health centers (34), Dubai Health is characterized by a pillar related to philanthropy namely: “giving.” In addition to sharing its goals with Dubai Health, as illustrated in Figure 1, MBRU aligns its efforts nationally with the Ministry of Higher Education and Scientific Research- Outcome-based Evaluation Framework (35) and internationally with the United Nations- Sustainable Development Goals (SDGs) (36, 37).

College of Medicine (CoM) at MBRU offers a Bachelor of Medicine and Bachelor of Surgery program (MBBS). The MBBS curriculum, which spans over 6 years, is prudently structured into three distinct phases (Phases 1, 2, and 3), where each phase is designed with an integrative curriculum that adopts a spiral curriculum (38). This andrological approach ensures that key foundational concepts are revisited and reinforced at increasing levels of sophistication throughout the program, as the students gradually progress in their medical training. In Phase 1, covering Year One, the educational focus consists of building core medical knowledge, achieved through delivering courses such as anatomy, biochemistry, and physiology with early clinical exposure. Phase 2, covering Year Two and Year Three, begins with the transition into clinical practice, emphasizing systems-based pathologies in preparation for Phase 3 (i.e., year 4 through year 6), which is entirely clinical. During the last phase, the medical students receive hands-on clinical training across various specialties in the allocated teaching hospitals (39). The andrological approaches and assessment formats, within the medical college and at the assigned teaching hospitals, vary and evolve, alongside the progressive spiraling curriculum.

The ethos of assessment processes at MBRU are based on fairness, reliability, and validity, with continuous feedback mechanisms put in place to ensure that all students are evaluated and supported in their progression (40). These processes are governed by rigorous and transparent policies delineated by an assigned college-level committee, commonly referred to as the Students Assessment and Progression Committee (SAPC). At the time of the study, the corresponding committee at CoM comprised six faculty members- 3 who were involved in learning and teaching only, and 3 who held key administrative positions along with their teaching duties. They convened regularly, 4 times a year. They were entrusted to oversee the planning, implementation, and evaluation of the MBBS assessment method, including but not limited to: formats, designs, and results, that was put in place as part of the respective program. The assessment development workflow involved collaborative input from subject matter experts (academic faculty and clinical educators) who were in charge of generating questions in alignment with the learning outcomes (41). The questions are compiled and then peer-reviewed by the respective committee members to ensure the attainment of a high-quality assessment method. In Phases 1 and 2 of the MBBS, the assessment method was designed to incorporate a balanced assortment of formative and summative assessments, including Multiple-Choice Questions (MCQs), Objective Structured Practical Examinations (OSPEs), and Objective Structured Clinical Examinations (OSCEs) (42).

Among the homegrown learning and teaching interventions integrated into the MBBS is a research module comprising five interconnected courses. This module is delivered over the first five consecutive semesters of the MBBS, where each of the academic years of Phases 1 and 2 comprises two semesters (i.e., the module is delivered over two and a half academic years) (43). These courses are integrated into the respective curricula to enhance students’ understanding of epidemiology, biostatistics, and research methodology. The first three module courses focus on building foundational knowledge in epidemiology and biostatistics, while the final two module courses provide students with the opportunity to apply their learning by undertaking independent research projects under the supervision of CoM in-house and adjunct faculty members (43). This research module has been continuously developing through design-based research (44, 45) and in alignment with the complementarity between Kolb’s Experiential Learning Theory (46), complemented by social constructionism theory (47, 48).

At the time when the data of the current research work was collected (academic year 2017–2018), MBRU was a newly established university. Two cohorts had been enrolled, with the inaugural intake starting in academic year 2016–2017 and the second one starting in the following academic year. In the academic year 2017–2018 (when the data of the current research work was collected), a total of 42 students were enrolled in Year One and 45 students in Year Two of MBBS at MBRU [77% of whom were female (and the remaining were male) and 33% were UAE nationals]. These students were from 26 nationalities.

### 2.2 Research design

A convergent mixed methods study design was employed to gain a systemic understanding of medical students’ perception of assessment and its impact on their learning. Quantitative and qualitative data were concurrently collected and analyzed. Merging the findings from the two sources was done to raise the validity of the study’s findings, and relied on the iterative Pillar Integration Process (PIP) which generated a joint display model (49). This technique provides a structured approach to combining different data types, facilitating a deeper understanding of the research topic (50).

This convergent mixed methods design was based on two undergraduate medical student-led projects as part of the experiential learning segment of the abovementioned five-course research module that is integral to MBBS (43). This happened through peer collaboration with one student engaging in the quantitative element and the other handling the qualitative aspect of the study. It is worth highlighting that these student-led projects were part of the first round of implementation of the respective five-course research module.

Those two arms of the study were approved independently by the Mohammed Bin Rashid University of Medicine and Health Sciences-Institutional Review Board (MBRU -IRB -SRP2018 -010 and MBRU-IRB-SRP2018-009), as per the abovementioned five-course research module requirements.

**FIGURE 2:**
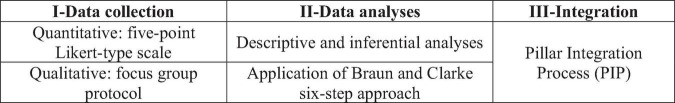
Visual representation of the current multi-phased convergent mixed methods study.

In its entirety, the research project covered three phases (Figure 2). FE was responsible for carrying out the quantitative part, whilst EK lead the qualitative components of the current research work.

Phase I of the current research work involved the development of two data collection tools for the respective independent studies/data collection initiatives. The analyses and identification of key findings were conducted independently in Phase II. The quantitative data was analyzed through descriptive and inferential analyses. As for the qualitative component, the Braun and Clarke six-step approach (51) was employed to inductively analyze the data. This multi-staged approach to thematic analysis is commonly deployed in research around health professions education (52). NVivo software version 12.0 plus (QSR International Pty. Ltd., Chadstone, Australia) (53) was relied on to assign codes to the categories and themes, and in turn, facilitate the categorization of the text fragments highlighted by the data analyzers. During Phase III of the current research work, the PIP was utilized to integrate the findings of the quantitative and qualitative analyses, and meta-inferences were generated (49). PIP involves four stages: listing, matching, checking, and pillar-building, with the goal of identifying meta-inferences (50, 54, 55).

### 2.3 Data collection

**FIGURE 3:**
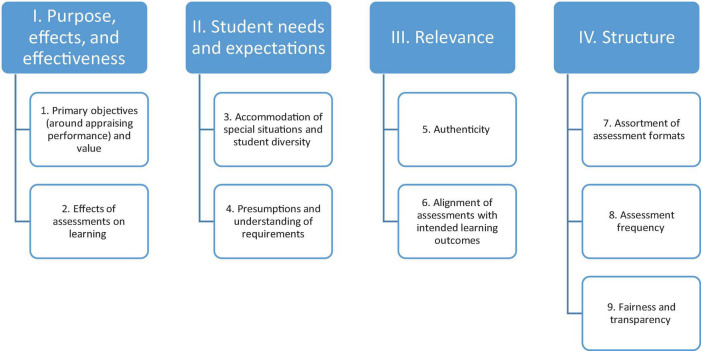
Affinity diagram underlying the quantitative and in turn qualitative tool development.

All the data was collected over a span of 2 months from March to April 2018. The quantitative data was collected through a tailormade survey protocol of nine subsections (Supplementary Appendix 1), and the qualitative data was collected through a tailormade focus group protocol of four subsections (Supplementary Appendix 2). The quantitative tool included nine subsections. These subsections were devised by the researchers who based their opinions on established guidelines (56, 57) and several existent classifications of assessment characteristics (58–61), followed by a series of discussions among them. As outlined in Figure 3, these nine subsections of the quantitative data collection tool (i.e., 28 components of five-point Likert-type scale across the nine subsections) were inductively classified into four segments that formed the basis of the qualitative tool (i.e., focus group protocol composed of four segments).

Both protocols underwent two validation phases. Firstly, two faculty members at CoM and one staff member at the Strategy and Institutional Excellence unit at MBRU were engaged in the content validity. Secondly, the questions of the data collection tool were discussed with two medical students to assess clarity, comprehensibility, and readability of the questions and the flow by which they were presented (i.e., face validity). The confidentiality of the participants was maintained throughout the research study, and no personal identifiers were recorded.

### 2.4 Quantitative component

The quantitative component of the current multi-phased research work comprised a cross-sectional observational survey design where a tailormade survey with a five-point Likert-type scale was administered via the online Google Forms platform to all 87 undergraduate medical students in Year One and Year Two of MBBS at CoM. The survey consisted of two parts: (i) demographic characteristics of the participants (namely: which MBBS cohort the participants belonged to, and their gender and their age range) and (ii) perception of the appropriateness of nine domains of assessment method characteristics (58–61):

1. Primary objectives (around appraising performance) and value.
2. Impact of assessments on learning.
3. Accommodation of special situations and student diversity.
4. Presumptions and understanding of requirements.
5. Authenticity.
6. Alignment of assessments with intended learning outcomes.
7. Assortment of assessment formats.
8. Assessment frequency.
9. Fairness and transparency.

The latter part of the survey relied on a five-point Likert-type scale (1: Strongly Disagree, 2: Disagree, 3: Neutral, 4: Agree, and 5: Strongly Agree).

Participation was confirmed in this voluntary study through a mutual agreement via an enclosed informed consent form that appeared on their screens prior to filling out the electronic survey. Since the survey was self-administered and filled out, interviewer and assistant bias was avoided and there were no confounding factors. The participants needed 7–10 min on average to fill out the respective survey.

### 2.5 Qualitative component

The qualitative data was collected through a series of focus group sessions. The purpose of the focus group sessions was to explore student perception of assessment and its impact on their learning.

This exploration relied on a pre-set tailor-made focus group protocol (Supplementary Appendix II) composed of four segments that were, as previously illustrated in Figure 3, based on an inductive categorization of the nine domains identified for the quantitative tool:

1. Purpose, effects, and effectiveness: motivations behind the assessment, its outputs, and its ability to achieve its intended results.
2. Student needs and expectations: elements of assessment preparation that students perceive as necessary to enhance their performance, as well as the tools educators can provide to aid in the preparation (while accommodating special needs).
3. Relevance: alignment of assessments with the learning objectives, and the congruence between assessment and learning.
4. Structure: various elements that contribute to the arrangement of assessment including but not necessarily limited to assessment design, format, frequency, reliability, equity, and inclusivity.

Each focus group session was held over the span of 60 min. Each segment of the protocol was allocated 15 min of discussion.

A complete list of enrolled students was obtained from Student Services and Registration unit at MBRU. As per established guidelines for conducting focus group sessions (62), a total of 10 students were randomly selected from each cohort and in turn invited to participate in the respective focus group sessions. Out of the 10 invitees of Year One, 4 students showed up. As for invitees of Year Two, all 10 students participated. To maintain balance which is recommended when more than one focus group session are implemented in the same research study (31, 63), in terms of number of participants, across the focus group sessions, Year Two participants were divided into two groups consisting of five participants each. As such, three focus group sessions were conducted, one corresponding to Year One, and the other two corresponded to Year Two.

The focus group sessions were conducted in MBRU in private study rooms. The focus group protocol was used as a tool to lead discussion amongst the participants, and their interactions were audio recorded for transcription.

The study investigators were not present during the sessions. The discussion facilitators were MBRU staff members (one assigned to each focus group session) who were neither directly involved in the learning and teaching (including assessments), nor investigators of the current study, with one facilitator present per focus group session. The respective facilitators were responsible for ensuring that the sessions ran smoothly and in a timely manner. Prior to the session, one of the study investigators met with the three facilitators to form a common ground and ensure consistency throughout the assigned facilitation.

The facilitators were briefed regarding their role, and provided with the focus group protocol to review and to ensure full comprehension of the purpose of the focus group session. Specific instructions to the facilitators were expressed verbally during the briefing session. These instructions included asking the facilitators to minimize interfering in the content of the discussions by avoiding sharing individual opinions during the sessions and ensuring that every participant in the group expresses their opinion. Facilitators were also advised on how and when to use probes. Each participant was assigned a unique identifying number. Each identifying number included a serial number (1–14) preceded by the letter “S,” as well as an indication of the corresponding cohort (Year One and Year Two).

### 2.6 Data analysis

#### 2.6.1 Quantitative analyses

The quantitative data was analysed using the Statistical Package for Social Sciences (SPSS, version 27.0).

The descriptive analysis constituted of computing an overall score of “Current assessment method efficacy” tool (i.e., across the 28 components of the five-point Likert-type scale composed of nine subsections), along with a score for each of the nine subsections of the tool. Then, the mean and standard deviation for each of the components and the scores: overall and for each of the nine subsections of the tool, were then calculated. Following that, the percentage of the mean for each of the components [dividing the respective mean by five (since it is the maximum possible value) and multiplying it by 100] and for each of the scores [dividing the respective mean by (total number of components multiplied by five) (i.e., the maximum possible value) and multiplying it by 100] were calculated, which determines where the corresponding values lie on the scale, with the following cut-offs according to established interpretation recommendations (64–66): 0%–6.25% = Strongly Disagree, 18.75%–31.25% = Disagree, 43.75%–56.25% = Neutral, 68.75%–81.25% = Agree A, and 93.75%–100% = Strongly Agree SA.

The validity tests of Cronbach’s Alpha and the Principal Component Analysis (PCA) were performed to ensure the internal consistency and check external variance, respectively, of the adapted tool. To select the appropriate comparative analyses tests, the Shapiro–Wilk test of normality was conducted for the overall score. Since the data turned out to be not normally distributed, the Mann-Whitney test was used to compare the overall score between female and male students, and the overall score between the two cohorts (Year One and Year Two). Finally, Kruskal-Wallis test was used to compare the overall score between the three age ranges (under 18, 18–24, and 25–33).

#### 2.6.2 Qualitative analysis

After completion of data collection, the data was manually transcribed and analyzed thematically by four researchers (EK1, FO, EK2, and RL). The subjectivity of the researchers was recognized up-front so as not to impact the integrity of the thematic analysis. One of the data analysers (F.O.), who has developed expertise in conducting qualitative socio-behavioral research, assured the rigor of the deployment of the analytical framework through ensuring the consistent application of the underpinning assumptions. By displaying rather than avoiding the investigators’ orientation and personal involvement in the research and by evaluating interpretations according to their impact on participants, investigators, and readers (67), the quality control exercised in this investigation shifted from the objective truth of statements to understanding by people. The data was divided into three datasets, one for each of the focus groups.

The analysis was inductive based on a constructivist epistemology. This expositional approach aided in providing an in-depth understanding of the subject of investigation. The analysis followed the six-step approach introduced by Braun and Clarke (51). This systematic approach to thematic data analysis has been highly supported in the field of healthcare education research (68).

The first step involved the four researchers familiarizing themselves with the dataset. The data was segmented into meaningful statements that relate to the overall purpose of the current research work and the corresponding research question. The datasets from the three focus groups were reviewed separately at first since the researchers were intentional about remaining open to observing potential difference(s) across the groups.

In the second step, the text fragments referring to the same aspects of student perception were clustered together and placed under one broad, all-encompassing label and the initial codes were generated. This process was performed for each of the three separate datasets.

The data was examined line-by-line, until data saturation was attained.

The multiple ways in which the concepts cross-over and relate to one another were identified. This allowed for the generation of categories which covered all the meaningful themes available in the dataset.

This process enabled the researchers to move into the third step of the approach, wherein the categories were examined to identify the best approach to merging them into overarching themes.

**FIGURE 4:**
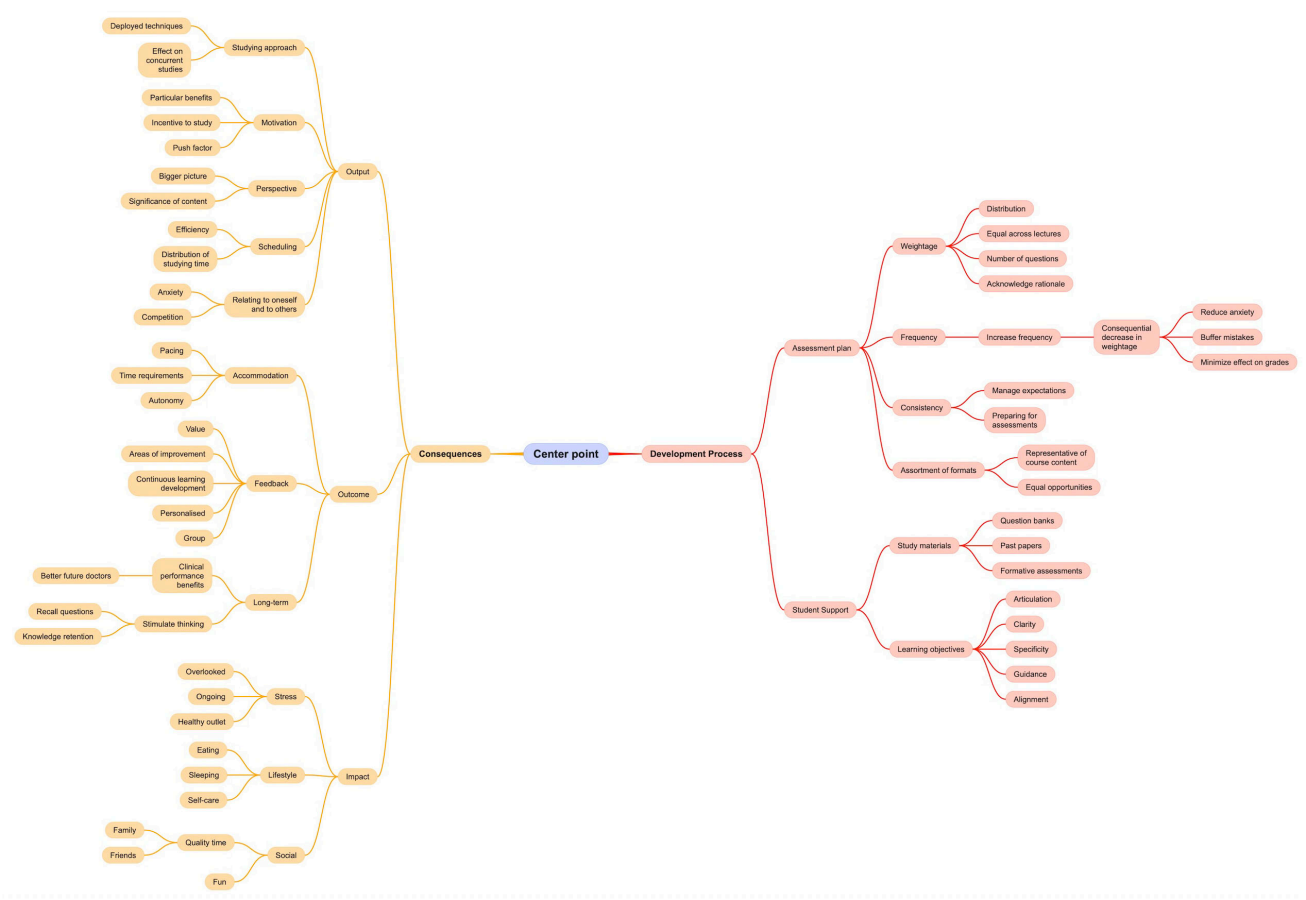
Mind map deployed as a tool to facilitate the qualitative analysis. This figure is meant to demonstrate a steppingstone in the analysis process. The output of respective analysis is Figure 4.

In the fourth step, the generated themes and categories were reviewed to ensure data grouping was valid and there was sufficient difference between the clusters to warrant segregation (Figure 4).

To complete stage five, all themes and categories were coded and in turn defined in the context of the study. A respondent validation followed, where all 14 participants were invited to a virtual discussion (10 of whom actually connected). In this meeting, one researcher (FO) guided attendees through the qualitative research questions, analysis process, and the generated conceptual model. After considering how well their responses resonated with the generated model, the attendees agreed with all the identified codes and how the model portrayed the connections between categories and themes. The sixth and final step of the approach included putting together a thorough report of the study findings.

#### 2.6.3 Information integration

The findings from the quantitative and qualitative analyses were merged to come up with meta-inferences using the iterative joint display analysis methodology (69). The findings generated from the two datasets were compared, and areas of similarity and differences were highlighted. The researchers also examined the data for findings that did not overlap between the two datasets and were unique to one of the two independent analyses. Through the integrative process (as opposed to pure triangulation), the researchers were able to emphasize key findings from both datasets, identifying areas of corroboration, as well as discordance. In other words, integration or merging of findings allows for going beyond looking at where the findings from different data sources confirm each other (i.e., triangulation) to portraying areas where the findings from different sources refine or expand overall understanding, or even contradict each other (70). Accordingly, the integration of the two sets of findings allowed the researchers to develop a holistic understanding of student perception of the assessment method and its consequences.

## 3 Results

The current research work adhered to the guidelines of reporting on mixed methods research (31). Accordingly, the analysis of the quantitative data addressed the first research question of the current study, while the output of qualitative analysis answered the second research question. Furthermore, as previously mentioned in the Methodology section, the third research question was addressed through the integration of quantitative and qualitative findings.

### 3.1 Quantitative results

A total of 60 students completed the survey instrument on Google forms platform. The response rate was 65% for Year Two students and 53% for Year One students, out of which 78.3% were female students and the rest were male. Out of the 60 students, three were under 18, 56 were between 18 and 24, and one above 24. The reliability score of Cronbach’s Alpha for the tailor-made evaluation tool that captured the stakeholders’ perception was 83.5%. The percentage of the total average of agreement of current assessment method efficacy was 62.24%, as per Table 1.

**TABLE 1:** Output of descriptive quantitative analysis.

Survey subsection	Component	Mean (SD)	Percentage of the mean*	Category	Scores of survey subsection		
					Mean (SD)	Percentage of the mean*	Category
Primary objectives (around appraising performance) and value (3 components)	I think that the assessment method is helping me in my phase progression	3.43 (0.87)	68.6%	N-A	8.95 (2.15)	59.67%	N-A
	I think that the weightage assigned to the various assessment formats (TBL, MCQs, SAQs, OSPEs, and OSCEs) is reasonable	2.75 (1.08)	55%	N			
	I feel that the current assessment method effectively assesses my knowledge, skills, and attitudes	2.77 (0.98)	55.4%	N			
Impact on learning (2 components)	The assessments highlight key concepts which improve my learning by bringing to my attention my strengths and weaknesses	3.22 (1.03)	64.4%	N-A	6.45 (1.88)	64.5%	N-A
	The assessments are consistent with my learning objectives	3.23 (1.06)	64.6%	N-A			
Accommodation for special situations and student diversity (3 components)	I feel that I am counseled enough regarding exams	2.35 (1.07)	47%	N	7.82 (2.69)	52.13%	N
	Under performing students are provided with improvement opportunities	2.60 (1.14)	52%	N			
	The academic advisor is helpful in resolving assessment-related issues	2.87 (1.20)	57.4%	N-A			
Presumptions and understanding of requirements (2 components)	I am made aware of the assessor(s) expectations of my performance ahead of assessments	2.53 (1.11)	50.6%	N	5.65 (1.65)	56.5%	N-A
	The difficulty level of assessments is appropriate to my knowledge and skills	3.12 (0.94)	62.4%	N-A			
Authenticity (2 components)	The assessment content mirrors real life situations	3.20 (1.12)	64%	N-A	6.87 (1.62)	68.7%	N-A
	The assessment formats (especially OSCEs and OSPEs) assess skills that are close to clinical practice	3.67 (0.91)	73.4%	A			
Alignment of assessments with intended learning outcomes (1 component)	Assessments are aligned with course objectives	3.27 (1.16)	65.4%	N	3.27 (1.16)	65.4%	N-A
Assortment of assessment formats (7 components)	I think that the TBL constitute a useful assessment format to evaluate my learning	4.05 (0.77)	81%	A	24.65 (3.57)	70.43%	A
	I think the TBL are helping me to comprehend principles of effective teamwork	3.62 (1.24)	72.4%	A			
	I think TBL are fostering my engagement with my colleagues	3.72 (1.03)	74.4%	A			
	I think that the MCQs constitute a useful assessment format to evaluate my learning	3.35 (1.29)	67%	N-A			
	I think that the SAQs constitute a useful assessment format to evaluate my learning	2.87 (0.97)	57.4%	N-A			
	I think that the OSPEs measure skills and competency in an excellent way	3.30 (1.03)	66%	N-A			
	I think that the OSCEs measure skills and competency in an excellent way	3.75 (0.95)	75%	A			
Assessment frequency (2 components)	I believe that the assessments are spread in an optimal manner throughout the academic year	2.52 (1.21)	50.4%	N	5.75 (1.95)	57.5%	N-A
	I think that the frequency of assessments is fair	3.23 (1.29)	64.6%	N-A			
Fairness and transparency (6 components)	The assessment formats consider the different learning techniques of the students	2.83 (1.04)	56.6%	N-A	17.73 (4.17)	59.1%	N-A
	The assessment plan and system of grading were shared ahead of the assessments	3.35 (1.22)	67%	N-A			
	Feedback on assessments has been timely	2.28 (1.14)	45.6%	N			
	Feedback on assessments has been helpful	3.43 (1.21)	68.6%	N-A			
	Feedback from students to modify future preparations of assessments have been taken	2.85 (1.21)	57%	N-A			
	Students are oriented with new assessment formats ahead of time	2.98 (1.08)	59.6%	N-A			
Overall (28 components)					87.13 (13.13)	62.24%	N-A

N, Neutral (43.75%–56.25%) and A, Agree (68.75%–81.25%). TBL, team-based learning; MCQs, Multiple-Choice Questions; SAQs, Self-Assessment Questionnaires; OSPEs, Objective Structured Practical Examinations; OSCEs, Objective Structured Clinical Examinations.

*Percentage of the mean for each of the components [dividing the respective mean by five (since it is the maximum possible value) and multiplying it by 100] and for each of the scores [dividing the respective mean by (total number of components multiplied by five) (i.e., the maximum possible value) and multiplying it by 100] were calculated, which determines where the corresponding values lie on the scale, cut-offs according to established interpretation recommendations.

According to the PCA (Kaiser-Meyer-Olkin Measure of Sampling Adequacy), 56.3% of the variance can be explained by the instrument, which means the instrument is not only reliable but also, according to Bartlett’s Test of Sphericity, valid to measure what it is intended to measure (*p* < 0.001). Along the same lines, the Bivariate Spearman Correlations showed how changes in the components could explain the changes in the scores (overall and that of each of the nine subsections of the survey).

There was no statistical significance between the two cohorts, when it comes to the overall score of agreement. Among the subsection scores, the only exceptions were Assortment of Assessment Method, and Fairness and Transparency. Students of Year One, with a mean of agreement of 25.33 (3.45), rated the Assortment of Assessment Methods higher than Students of Year Two, with a mean of agreement of 23.38 (3.53). As for the Fairness and Transparency, Students of Year One, with a mean of agreement of 19.29 (4.11), rated their experience significantly higher than Students of Year Two, with a mean of agreement of 16.90 (3.99).

Also, there appeared to be no statistical significance between female and male students, neither in relation to the overall score nor to any one of the subsection scores.

Finally, in relation to the overall score, students between ages of 25 and 33, with a mean of 104, rated the experience significantly higher than those under 18 [104 (6.56)], followed by those between 18 and 24 years of age [85.93 (12.7)]. As for the subsection scores, all revealed no statistical significance across the age groups, except for Fairness and Transparency, where students under 18, with a mean of 23 (2.65) rated the experience significantly higher than those between 25 and 33 (with a mean of 22), followed by those between 18 and 24 years of age [17.38 (4.05)].

### 3.2 Qualitative results

**FIGURE 5:**
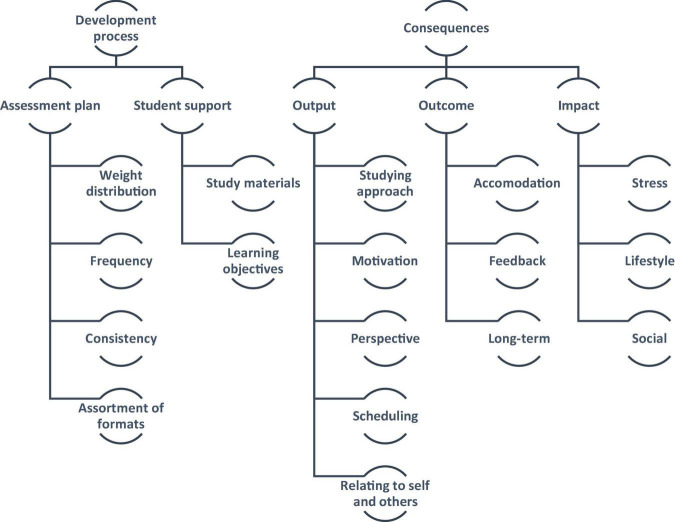
The study’s conceptual framework: “medical students” take on assessment methods’.

The sample included a total of 14 participants; 4 participants belonged to Year One and 10 participants belonged to Year Two. The inductive thematic analysis yielded, as per the study’s conceptual framework: “Medical Students” Take on Assessment Method’, two overarching themes: Development Process and Consequences. Within the Development Process theme, two categories emerged: Assessment plan and Student support. As for the Consequences theme, it included three other categories: Output, Outcomes, and Impact (Figure 5).

#### 3.2.1 Theme 1: development process

This theme refers to how the students tend to perceive the assessment method as an unfolding of events. This theme includes the students’ reflections on the elements that they consider characteristic of the assessment method in place.

##### 3.2.1.1 Assessment plan

This category refers to the students’ reflections on the characteristics that are believed to be relevant to the development of the assessment method.

The participating students expressed some concerns regarding the distribution of weightage in their assessments, in terms of fairness and also clarity of communication around the subject matter. Many students stated that they would prefer an assessment that gave equal importance to all lectures with an equal number of questions per lecture. They also felt that the total number of questions in an exam should be determined based on the weightage of the exam. However, students did acknowledge that this rule may not always apply as the degree of importance across different lectures can vary, particularly as students slowly progress from the basic sciences to clinical knowledge and application.

Y2S1: “…so fairness in a test would probably be an equal distribution of questions per lecture. So, for example, if we have 10 lectures and 10 questions, I expect a question from each lecture…”

Y2S2: “…maybe one lecture carries more importance or has not been tested before so maybe it could carry more questions…”

One suggestion that some students raised is to diffuse the grading weight of each assessment by increasing their frequency. This, in turn, would lighten the consequences of making mistakes and would reduce the anxiety that comes with taking exams. This, the students believe, would also encourage learning from mistakes.

Y1S6: “…it gave us a lot more opportunity to make mistakes and understand why we made those mistakes without having a huge drop in our grade point average…”

Participants also highlighted the importance of consistency across assessments. Some students pointed out that assessment styles varied a lot, which made managing one’s expectations and in turn preparing for the assessments more challenging.

Y2S8: “…I think it is different and there is no pattern for exams. Some exams are all memorization, some exams are all cases…”

A lot of students highlighted inclusiveness through recommending the integration of various assessment formats to ensure that study material can be tested appropriately and to give different learners equal opportunity to perform well.

Y2S10: “…certain things are better for testing in some subjects than others and I think integration is the best thing where you need a bit of both… There are different formats for a reason, they complement each other.”

##### 3.2.1.2 Student support

This category pertains to the students’ perception of the materials provided to them by the educators to aid in studying and preparing for assessments. The importance of preparing well for an assessment was brought up in the discussions amongst the students. The students listed several ways in which the educators could contribute to facilitating the process of preparing for an assessment. Students highlighted the usefulness of providing them with question banks, past papers, and formative assessments to practice for the summative assessments.

Y2S3: “…I think what we need is a bank of questions to practice normally so that we can get used to it.”

Another factor that the students felt was crucial was related to the quality of the learning and teaching, where they recommended raising the clarity of the learning objectives and also ensuring good conveyance of content (including but not limited to constructive alignment). The students emphasized that the more specific the objectives, the more refined the guidance. They also pointed out the importance of making sure that the assessments are effectively mapped onto the learning objectives.

Y2S4: “…The objectives actually talk about important fundamental basic information that you should get after finishing this lecture…”

Y2S5: “…I think fairness. if the test actually fulfils the objectives of the course or the material covered.”

#### 3.2.2 Theme 2: consequences

This theme encapsulates the students’ reflections on the effects of the deployed assessment method. The students tapped into what they perceived as results of the experiences with assessments. Apparently, some of these results immediately materialized. The students also brought up results that they believed will take time to be realized.

##### 3.2.2.1 Output

This category refers to the students’ thoughts pertaining to what they experienced while going through the assessment and/or soon afterward. This includes how assessments directly affect the students’ approach to studying, the various study techniques they employ around the assessment, and how these factors affect their concurrent studies. Some students found that a particular benefit to assessments is that they provide a reason or a push factor for certain types of students to study.

Y2S7: “…some people do not study unless they have something to study for like a deadline…”

However, all students agreed that during assessments, they find themselves inclined to studying simply for the sake of passing the assessment. This results in inefficient studying techniques and an inability to retain information for a long duration of time.

Y2S9: “…I know a lot of people who study only for the exam…they will forget everything by the time the exam finishes…”

Many students also tend to lose sight of the bigger picture, focusing on minor details that could come in the exam as opposed to the greater implications and significance of what they are learning.

Y2S1: “…I can ignore a pathophysiological process because I want to memorize a sentence that is marked in red…”

Apparently, students are often riddled with anxiety around the exam period, dedicating more time to studying and preparing for the respective assessment, often at the expense of the rest of their studies.

Y2S2: “…people are studying for the assessments in class while the instructor is teaching us something else…”

Y2S5: “…when I have a test in say 2 h, even if I am not studying, I am stressing. Mentally, I am not in the class…”

A few students also pointed out the potentially unhealthy competition that assessments can create amongst peers.

Y1S3: “…exams are kind of a way to create unhealthy competition. Is it helping me grow or is it helping me dislike others?”

##### 3.2.2.2 Outcome

This category highlights the students’ perception of the influence on learning of the various assessment approaches and feedback styles. From a temporal perspective, the students seem to consider the outcome to be occurring after the output (i.e., preceding category of the same theme).

A few students pointed out that the existence of assessments creates a pacing that is not necessarily fit for all students. It was highlighted that some students require more time to integrate the competencies they are meant to acquire, where the existence of a set timeline, punctuated with assessments, limits their autonomy in terms of pacing.

Y2S5: “…it is just not very fair to start off saying okay, this is a lecture. You have 2 weeks to study for an exam. We do not all learn at the same pace. We are different people, we learn differently…”

Students frequently alluded to how assessments lose a lot of their value if they are not accompanied with adequate feedback. Feedback is required to pinpoint areas of improvement and to support students in learning from their lived experiences, in general, and more specifically past mistakes.

Y2S1: “…if you do not tell me why I got it wrong, how are you expecting me to get it right again in the finals? so there should me more feedback on the questions.”

Students also favored personalized and detailed feedback. This can be complemented by group feedback so that students can maximize the learning.

Y2S2: “…I do not really like group feedback because we only discuss the questions the majority of the students got wrong. I could have gotten none of those wrong and still lost a lot of marks but I would not know where I went wrong…”

In terms of perceived long-term benefits of assessments, students emphasized the importance of assessments tackling clinically relevant topics.

Y1S3: “…what is the relevance of me knowing that this thing happens this way? …where is the line between learning to ultimately improve my performance as a future doctor versus learning for the purpose of passing an exam? …”

Students also emphasized that assessments should avoid simply testing direct recall, and that they should focus on stimulating thinking and assessing a student’s understanding of a topic.

Y2S6: “…we do not want questions where we can easily restate a piece of information… it is not testing acquired and integrated knowledge, it is in fact testing memorization…”

Y2S5: “… a lot of the questions could be helpful if they were framed in a way that encourages us to think more…”

##### 3.2.2.3 Impact

This category highlights students’ perception of how assessments affect students intra- and inter-personally.

There appeared to be a consensus among students regarding the impact of assessments on their personal lives in terms of their wellbeing: mental, physical, and social. All students emphasized how stressful assessments can be and the anxiety that comes along with needing to perform up to a certain standard. In fact, many feel that this aspect of their studies is overlooked.

Y2S6: “…It causes a lot of stress…”

Y2S4: “…I do not mean this in a selfish way, we are supposed to take care of people but are we sufficiently taken care of.”

Many students felt that their mental health issues are unseen and ignored, and many expressed their desire for a healthy outlet. Some students raised concerns regarding how their mental health can directly impact their academic performance. All the students agreed that having a specialized mental health professional would assist them in learning how to cope in better ways and lead happier lives.

Y2S3: “…as medical students, we are prone to depression. I am not saying we need someone to take care of us but we all handle things differently and it reflects in our performance…”

Y2S6: “I think the most important thing we need here is a psychologist or a guidance counselor who is experienced with dealing with medical students specifically.”

Students also pointed out that the period before assessment really affects their lifestyle and health. Many students develop disruptive eating and sleeping habits, spend long hours on their desk staring at a computer screen, and find that there is little time for self-care.

Y1S3: “…you stop talking to some people…you start eating differently, you start sleeping differently…”

Lastly, some students were particularly distressed by assessment’s impact on their social relationships with friends and family alike. Many students find it difficult to manage their time between work and fun, and miss out on spending quality time with their loved ones.

Y2S3: “…I spend less time with them…it should not be that way…” Y1S1: “…my social life is non-existent…”

### 3.3 Information integration

**FIGURE 6:**
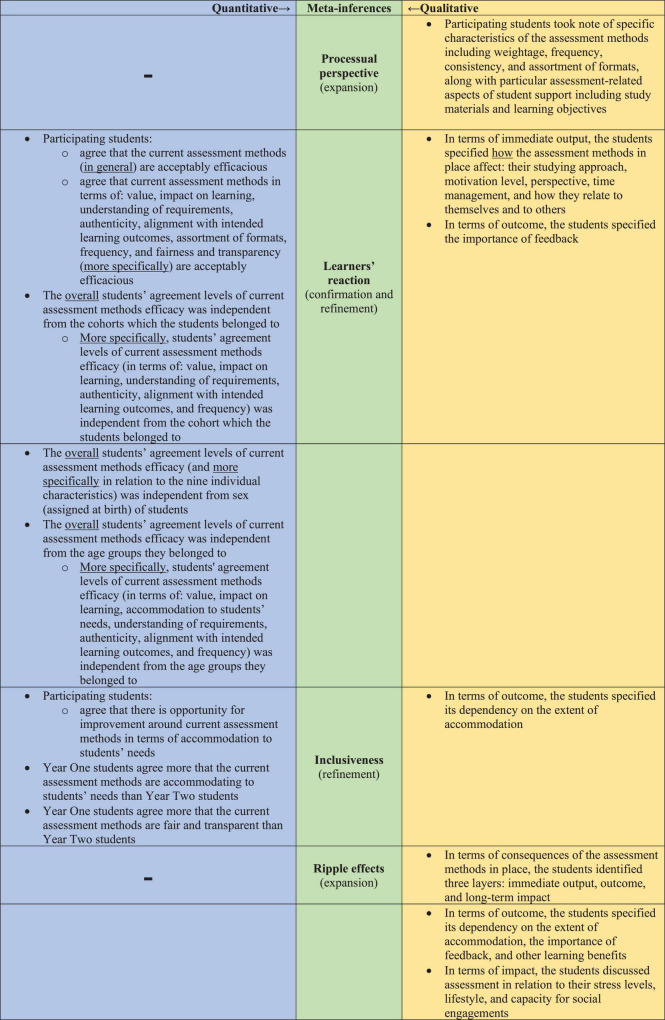
The iterative side-by-side joint display analysis process (of integrating the data findings) resulted in four meta-inferences: processual perspective, learners’ reaction, inclusiveness, and ripple effects. The secondary color Green emerged by mixing the primary color Blue with the primary color Yellow (symbolizing the critical thinking that took place to generate the meta-inferences from the integration of two sets of primary inferences). The integration led to expansion, confirmation, and/or refinement of the researchers’ overall understanding of the subject matter.

Four meta-inferences emerged from integrating the data findings, as illustrated in Figure 6: Processual perspective, Learners’ reaction, Inclusiveness, and Ripple Effects.

Firstly, in terms of the Processual perspective, the qualitative findings showed how the students perceived the assessment methods as a process, characterized by particular attributes, rather than discrete events. There was no counterpart to this observation among the generated quantitative findings, and hence, the qualitative component of the current study led to an expansion of the overall understanding of the subject matter. Secondly, in terms of the Learners’ reaction, narratively, the students reflected upon how the assessment methods in place affect them, highlighting the importance of feedback in terms of maximizing learning. Along those lines, quantitatively, the students agree that the efficaciousness of the assessment methods in place is acceptable, in general and in relation to particular attributes more specifically. The quantitative analysis also showed that this observation is independent from cohorts which they belonged to, their sex (assigned at birth), and the age group that they belong to. Accordingly, for the second meta-inference, the integration led to both: confirmation and expansion. Thirdly, in relation to the Inclusiveness meta-inference, the qualitative analysis showed that students indicate the dependency of the outcome of the assessments’ methods (i.e., learning) on the extent of accommodation. Quantitatively, the participating students agree that there is opportunity for improvement around current assessment methods in terms of accommodation to students’ needs. Also, relative to Year Two students, Year One students seemed to agree more that the current assessment methods, on the one hand, are accommodating to students’ needs, and on the other hand are fair and transparent. Accordingly, the integration of findings led to refinement of the understanding around the subject matter. Fourthly, in relation to the Ripple effects meta-inference, the qualitative findings showed that the students identified three layers of consequences of the assessment methods in place: immediate output, outcome, and long-term impact. There was no counterpart to this observation among the quantitative findings, and hence, the qualitative component of the current study led to an expansion of the overall understanding of the subject matter.

## 4 Discussion

The current study investigated medical students’ perceptions of the assessment method employed in a medical school in Dubai, UAE. It highlighted the key aspects of assessments that seem to influence the learning value, as determined by the students. It is recommended for the identified aspects to be taken into account when designing assessment methods to enable students to maximize the learning value of assessments. The current study showed that students tend to view assessment as a process. They seem to be aware of their emotional reaction to the assessment method in place, and to perceive it to have ripple effects that move from immediate output to outcome, and in turn impact. The study also revealed that students tend to emphasize the importance of the inclusiveness of an assessment method. The findings of this study further reinforce the notion that assessments are not merely evaluative checkpoints but rather deeply embedded elements of students’ learning journeys (7, 8), which hold the potential of greatly influencing the cognitive, emotional, and behavioral dimensions of the learners’ lived experiences (71).

The processual perspective of the students is worth elaborating upon. Students tend to perceive assessments collectively as an ongoing element of their educational development rather than isolated, high-stakes events. This aligns with sociocultural perspectives, which emphasize the importance of dialog, trust, and formative interaction in assessments that support learning (72). Students participating in the current study reflected on aspects such as fairness of grading, distribution of questions, and alignment with learning objectives and lectures’ content, highlighting, in accordance with the extensive literature on the subject matter (73), that assessments constitute an integral part of student progression, embedded within a broader andragogical framework. Assessments serve as a means of appraising a student’s understanding of course material, as well as their ability to apply their knowledge in real life. Restricting assessment to “a measure of performance” may lead to overlooking on one front, its potential for learning, and for enabling and empowering the learners (71), and on the other front, its potential impact on mental health, and habits and lifestyles (74, 75). Majority of the students confessed that assessments are a source of stress and anxiety to them. The pre-occupation with assessment can also impact social relationships and daily habits. Many students admit that during periods of assessments, they find themselves isolating from social gatherings, forgetting to take care of themselves, and having disruptive sleeping habits. These lifestyle changes further exacerbate the state of their mental health during this time.

The process-oriented view observed among students in the current study supports the concept of Learning-Oriented Assessment (LOA). This concept was initially proposed by Carless (76), and emphasizes that assessments are designed not solely for performance judgment but also to foster learning. LOA positions tasks as learning opportunities, the centrality of timely and actionable feedback, and student engagement in criteria-setting (76). Students participating in the current study explicitly called for iterative assessments and opportunities to learn from mistakes. This view is supported by McMillan and Moore (77), who argue that a culture that allows for “being wrong” is foundational for fostering growth mindsets and self-regulated learning (52, 73), along with global citizenship (74, 77, 78). Such a tolerant culture reframes traditional assessment paradigms, suggesting that assessment should serve as a learning catalyst, not a terminal judgment mechanism.

Students’ desire for opportunities to err and to strive to improve performance and/or standing also underscores the importance of maintaining classroom environments that mitigate the fear of failure, welcoming formative feedback—a theme echoed in neurological research showing that mistakes can stimulate dopamine release and promote long-term learning (77). Participating students highlighted assessment weightage as a critical factor which can often limit their tolerance “to making mistakes (and in turn the potential of learning from/through them).” As per the quantitative component of the current study, the percentage of the mean of the following scale component: “I think that the weightage assigned to the various assessment formats [Team-Based Learning (TBL), MCQs, Self-Assessment Questionnaires (SAQs), OSPEs, and OSCEs] is reasonable” was 55%. The qualitative focus group responses showed that students felt that assessment weightage was often “too high,” which (according to the students) often leads to stress and anxiety (79, 80). Another highly relevant characteristic which revealed diverse opinions and corresponding rationales was the assessment frequency. The percentage of the mean of the following scale component: “I think that the frequency of assessments is fair” was around 65%. A variation in responses, however, was also observed in the focus group discussions. Some students preferred lower frequency of assessments as that was deemed to reduce stress: they would rather “get things over and done with, in one go,” “a once and for all” kind of attitude. Other students appreciated higher frequency, as it reduced the weightage per assessment and found it to help in tracking their performance. A similar observation regarding the perception of increased assessment frequency as a tool to aid in the learning process was made by Vaessen et al. (81).

A recurring subject, in the current study, was the emotional toll of assessments, particularly stress and anxiety. Many students reported heightened levels of pressure during exam periods, mirroring the findings from a multicenter study performed in the United Kingdom which linked the proximity of final exams with elevated anxiety and depression levels in medical students (82). These findings are consistent with broader literature on assessment-induced psychological strain (82–87), which shows that high-stakes testing environments often correlate with increased distress and also decreased performance in some learners, particularly when feedback is lacking or untimely. Furthermore, students reported feeling overwhelmed by assessment load and a lack of perceived autonomy, which aligns with findings from Coutts et al. (88), where high assessment demand appeared to significantly reduce intrinsic motivation, increase negative mood states, and encourage surface learning approaches. The emotional impact of assessments—particularly when stakes are high, and support is insufficient—emphasizes the need for institutional strategies that incorporate mental health resources and workload distribution planning (75, 88, 89).

The extent of inclusiveness of the assessment method in place was repetitively alluded to by the students. It emerged as a key concern among participating students, particularly in relation to fairness, diversity of assessment formats, and alignment with learning styles and needs. First-year students, in the current study, reported more favorable perceptions of fairness and transparency than their senior counterparts, potentially reflecting increasing curricular complexity and assessment expectations. This reinforces the need for adaptive assessment practices that evolve with students’ academic progression. Inclusive assessment design, informed by Universal Design for Learning (UDL) and inclusive andragogy, aims to provide equitable opportunities for all students by acknowledging diversity in learning preferences, cognitive processing, and life circumstances (90). Similarly, in other studies, students have expressed strong support for having choice in assessment formats (e.g., presentations, written assignments, and oral exams), as it enhanced their autonomy, performance, and satisfaction—particularly for students with Additional Learning Needs (ALN). Our findings also suggest a need for diverse assessment formats to better accommodate a larger number of learners. The quantitative findings reflected preference for certain formats such as TBL and OSCEs. The qualitative findings provided more depth pertaining to students’ views of the different assessment formats. Overall, students preferred case-based questions, as well as practical assessments such as OSCEs, indicating them as effective tools to assess their knowledge and understanding of the material. However, the students noted that a variety of assessment formats is necessary to assess different types of skills and to give equal opportunity for all learners. However, while the provision of choice supports well-being and self-determination, it must be carefully managed to ensure parity and rigor across modes (7). There is also an andragogical responsibility to scaffold skills development across diverse formats to avoid over-specialization or avoidance of essential competencies, such as presentation or communication skills. Importantly, institutions must support faculty in developing inclusive assessments through training and flexible administrative systems (8).

The consequences of the assessment method in place, according to the participating students in the current study, were happening at different layers of time. Students perceived assessments to have “ripple effects” that begin with immediate academic responses (e.g., strategic studying and isolation from other coursework), extending to behavioral changes, and culminating in long-term impacts on mental health, motivation, and peer relationships (74, 89). This echoes the findings of Lyndon et al. (86), which highlight that both the design and stakes of assessment influence not just academic performance, but also student psychological outcomes and professional identity formation (86). High-stress assessment environments were associated with competitive behavior, anxiety, and diminished peer collaboration, further reinforcing the need to redesign assessments for collaborative learning and mental well-being (89). These effects suggest that assessment practices should be continuously reviewed for holistic impact, not just on academic outcomes but also on the sociocultural and emotional dimensions of student life. Additionally, students voiced concerns about surface learning and reduced intrinsic motivation during periods of high assessment load—a pattern supported by research showing that clusters of assessments without feedback diminish perceived competence and engagement (88). Conversely, when feedback is timely and actionable, students report increased perceived competence and motivation, highlighting the need to embed structured, feedforward mechanisms into assessment cycles (76).

The current study, through its inductive qualitative analysis, introduced a novel conceptual framework, namely: “Medical Students” Take on Assessment Method’, that can be deployed by other learner-centric higher education institutions, in general, and more specifically medicine and health sciences universities to better understand how students characterize assessment methods. As such, this innovative framework can feed into curriculum design and corresponding investigations, and offers a student-centric lens for appraising assessment policies and practices. The study also introduced a data collection tool (five-point Likert-type scale): “Current assessment method efficacy,” which proved to be reliable and valid in the context of the study. This tool can be used by other similar medical schools to capture their students’ perception of the efficacy of the assessment method/extent of agreement regarding the appropriateness of the assessment method. This tool enables systematic evaluation of student sentiments in regard to assessment, and can serve as a valuable quality assurance mechanism for medical institutions seeking continuous quality improvement (91) be it via design-based research or otherwise (89) all of which contributes to sustainable development, in general, and specifically United Nations- Sustainable Development Goal 4: Quality Education (92). Within the context of this study, continuously improving health professions’ education eventually feeds into Sustainable Development Goal 3: Good Health and Wellbeing (93).

## 5 Limitations

The study has a few limitations. First, the generalizability of the findings is limited since the study took place in a single university of medicine and health sciences, including only two preclinical cohorts of medical students. The transferability of findings to similar institutions is quite strong, though, given the effective integration of quantitative and qualitative findings through the deployment of a mixed methods research design, along with the provision of a detailed description of the context of the respective study. Second, the study relied on self-reported data, which may be subject to response bias, in general, and more specifically, social desirability effects. Future research work could expand the sample size, compare and contrast findings across different educational settings, and integrate the findings with different sources (e.g., faculty perspectives and/or students’ performance). Additionally, longitudinal studies examining the effects (over time) of various assessment methods on student learning and well-being as they progress through basic and clinical medical sciences perspectives would provide further insights into the actual (as opposed to foreseen) outcomes and impacts of assessment (and not foreseen effects).

## 6 Conclusion

This study reinforced the importance of effectively assessing medical students’ competences while maximizing the learning value of the encapsulating assessment method. It showed that it is not about making choices around discrete aspects of assessment, but rather to consider assessment methods holistically as dynamic processes with lots of moving parts that ideally should be designed, implemented, and maintained in ways that would maximize its learning value, taking into account the context and the learners’ perception. The learners perceive assessment as a process which leads to immediate output, including but not limited to an emotional reaction, followed by outcomes and then long-term impacts. They genuinely care about its inclusiveness.

## Data Availability

The original contributions presented in this study are included in this article/Supplementary material, further inquiries can be directed to the corresponding author.
