# Supplementary material for: Medical students’ perception of assessment and its effects on their learning in Dubai: a convergent mixed methods study
Source: Front Med (Lausanne). 2025 Aug 4;12:1620437. doi: 10.3389/fmed.2025.1620437 (PMC12358476; doi:10.3389/fmed.2025.1620437)
Supplement: Supplementary file 1 [file Table_1.docx]

Supplementary Material

**Appendix I**

***Survey***

***Section 1: Demographic Characteristics***

**1. Please indicate your gender:**

⭘ Female

⭘ Male

⭘ Prefer not to say

**2. Please select your cohort:**

⭘ Year One

⭘ Year Two

**3. Please select the category that includes your age:**

⭘ Under 18

⭘ 18-24

⭘ 25-33

***Section 2: Survey Questions***

**Please select your level of agreement with the following statements:**

| **Component** | **Strongly Disagree****(1)** | **Disagree****(2)** | **Neutral****(3)** | **Agree****(4)** | **Strongly Agree****(5)** |
| --- | --- | --- | --- | --- | --- |
| **Primary objectives (around appraising performance) and value** | | | | | |
| - **I think that the assessment method is helping me in my phase progression** |  |  |  |  |  |
| - **I think that the weightage given to the various assessment formats (TBLs, MCQs, SAQs, OSPE, and OSCE) is reasonable** |  |  |  |  |  |
| - **I feel that the current assessment method effectively assesses my knowledge, skills, and attitudes** |  |  |  |  |  |
| **Impact on learning** | | | | | |
| - **The assessments highlight key concepts which improve my learning by bringing to my attention my strengths and weaknesses** |  |  |  |  |  |
| - **The assessments are consistent with my learning objectives** |  |  |  |  |  |
| **Accommodation for special situations and student diversity** | | | | | |
| - **I feel that I am counselled enough regarding exams** |  |  |  |  |  |
| - **Under performing students are provided with improvement opportunities** |  |  |  |  |  |
| - **The academic advisor is helpful in resolving assessment-related issues** |  |  |  |  |  |
| **Presumptions and understanding of requirements** | | | | | |
| - **I am made aware of the assessor(s) expectations of my performance ahead of assessments** |  |  |  |  |  |
| - **The difficulty level of assessments is appropriate to my knowledge and skills** |  |  |  |  |  |
| **Authenticity** | | | | | |
| - **The assessment content mirrors real life situations** |  |  |  |  |  |
| - **The assessment formats (especially OSCE and OSPE) assess skills that are close to clinical practice** |  |  |  |  |  |
| **Alignment of assessments with intended learning outcomes** | | | | | |
| - **Assessments are aligned with course objectives** |  |  |  |  |  |
| **Assortment of assessment formats** | | | | | |
| - **I think that the TBLs constitute a useful assessment format to evaluate my learning** |  |  |  |  |  |
| - **I think the TBLs are helping me to comprehend principles of effective teamwork** |  |  |  |  |  |
| - **I think TBLs are fostering my engagement with my colleagues** |  |  |  |  |  |
| - **I think that the MCQs constitute a useful assessment format to evaluate my learning** |  |  |  |  |  |
| - **I think that the SAQs constitute a useful assessment format to evaluate my learning** |  |  |  |  |  |
| - **I think that the OSPEs measure skills and competency in an excellent way** |  |  |  |  |  |
| - **I think that the OSCEs measure skills and competency in an excellent way** |  |  |  |  |  |
| **Assessment frequency** | | | | | |
| - **I believe that the assessments are spread in an optimal manner throughout the academic year** |  |  |  |  |  |
| - **I think that the frequency of the assessments is positively affecting my learning** |  |  |  |  |  |
| **Fairness and transparency** | | | | | |
| - **The assessment formats consider the different learning techniques of the students** |  |  |  |  |  |
| - **The assessment plan and system of grading were shared ahead of the assessments** |  |  |  |  |  |
| - **Feedback on assessments has been timely** |  |  |  |  |  |
| - **Feedback on assessments has been helpful** |  |  |  |  |  |
| - **Feedback from students to modify future preparations of assessments have been taken** |  |  |  |  |  |
| - **Students are oriented with new assessment formats ahead of time** |  |  |  |  |  |

**Appendix II**

***Focus group session protocol***

**Section I: Please share your thoughts about the purpose, effects, and effectiveness of the current assessment method (15 minutes)**

- **Primary objectives (around appraising performance) and value**
- **Effects of assessments on learning**

**Section II: Please share your thoughts about the student needs and expectations in relation to the current assessment method (15 minutes)**

- **Accommodation of special situations and student diversity**
- **Presumptions and understanding of requirements**

**Section III: Please share your thoughts about the ‘relevance’ of the current assessment method (15 minutes)**

- **Authenticity**
- **Alignment of assessments with intended learning outcomes**

**Section IV: Please share your thoughts about the ‘structure’ of the current assessment method (15 minutes)**

- **Assortment of assessment formats**
- **Assessment frequency**
- **Fairness and transparency**
